# Women's health: an imAging-based cardiovascular risK-rEdUction Program (WAKE UP) study. Rationale and design

**DOI:** 10.3389/fcvm.2025.1535827

**Published:** 2025-03-27

**Authors:** Samantha Wasniewski, Rafaella Kfouri Da Silva, Sofía Capdeville, Isabel Rivera Molina, Elena Virosta, Carolina Ortiz Cortés, Pablo Díez-Villanueva, Antonio Adeba, Marta Ruiz Lera, Lucía Muñoz, David Alonso, Eva García, Ruth Echeverría, Rocío Tarifa, Alessia Ferrarini, Josué Pagán, Jose Luis Ayala, Jorge Solis, Malissa Wood, Blanca Miranda, Luis Rodríguez Padial, Leticia Fernández-Friera

**Affiliations:** ^1^ HM CIEC MADRID (Centro Integral de Enfermedades Cardiovasculares), Hospital Universitario HM Montepríncipe, HM Hospitales, Facultad HM de Ciencias de la Salud de la Universidad Camilo José Cela, Instituto de Investigación Sanitaria HM Hospitales, Madrid, Spain; ^2^ Atria Clinic, Madrid, Spain; ^3^ Instituto de Investigación Sanitaria Hospital 12 de Octubre (imas12), Madrid, Spain; ^4^ Hospital Universitario Fundación de Alcorcón, Madrid, Spain; ^5^ Hospital Universitario La Princesa, Madrid, Spain; ^6^ Hospital Universitario Cabueñes, Asturias, Spain; ^7^ Hospital Universitario Marqués de Valdecilla, Santander, Spain; ^8^ Hospital Universitario HM Regla, León, Spain; ^9^ Complejo Hospitalario de Toledo, Castilla La Mancha, Spain; ^10^ Fundación Para la Investigación Biomédica del Hospital Universitario 12 de Octubre (FIBH12O), Madrid, Spain; ^11^ Centro Nacional de Investigaciones Cardiovasculares (CNIC), Madrid, Spain; ^12^ Electronic Engineering Department, Universidad Politécnica de Madrid, Madrid, Spain; ^13^ Center for Computational Simulation, Universidad Politécnica de Madrid, Boadilla del Monte, Spain; ^14^ Department of Computer Architecture and Automation, Universidad Complutense de Madrid, Madrid, Spain; ^15^ Hospital Universitario 12 de Octubre, Madrid, Spain; ^16^ CIBER de Investigaciones Cardiovasculares (CIBERCV), Madrid, Spain; ^17^ Department of Cardiology, Lee Health Heart Institute, Fort Myers, FL, United States; ^18^ Sociedad Española de Cardiología, Madrid, Spain

**Keywords:** women's health, cardiovascular disease prevention, vascular ultrasound, cardiovascular risk, lifestyle

## Abstract

**Background:**

Cardiovascular (CV) disease is the leading cause of death in women. Although 80% of CV disease events can be prevented, mortality is projected to increase, particularly in young women.

**Objectives:**

To promote CV health in women and encourage appropriate lifestyle changes by increasing awareness through vascular ultrasound imaging.

**Methods:**

WAKE UP is a prospective case-control study in a target population of 720 asymptomatic women, aged 40–70 years with ≥1 major CV risk factor (RF). Participants will attend a baseline visit and follow-up visits at 6 and 12-months. Each visit will include the assessment of traditional and non-traditional risk factors (age, blood pressure, weight, smoking, diet, physical activity, psychosocial aspects, reproductive factors, family lifestyle), CV risk scores [Fuster-BEWAT Score (FBS) and SCORE], perception of CV disease risk, blood sampling of hormones, lipids, glycemic metabolism, inflammation parameters and omics. At baseline visit, women will be randomized to undergo 2D/3D/strain vascular ultrasound (360 with imaging vs. 360 age- and RF-matched controls without imaging). Main outcomes will include changes from baseline to follow-up in overall knowledge, attitudes, and FBS.

**Conclusions:**

WAKE UP trial aims to raise awareness about womeńs CV disease and promote lifestyle changes. Imaging can play a key role by revealing the presence of atherosclerotic plaques in a directly relatable way and thus, larger effects are anticipated in women with plaques. WAKE UP can significantly impact CV prevention by involving innovative actions addressing a major public health need and by fostering complementary and synergistic actions.

## Introduction

Cardiovascular (CV) disease in women requires more attention, more research, and swifter action. It remains the leading cause of death in women, reaching 41% in Europe, 16 times higher than breast cancer (3%), corresponding to one woman deceased every minute (1, 2). The immense economic and social burden of this devastating disease is evident, including physical and psychological consequences for women and their families. Although 80% of CV events can be prevented, the trend is projected to worsen, with an alarming increase in death rate in women, especially at young age (3, 4). Nearly 50% of women aged 40–54 years have atherosclerosis, with ≥2 arterial sites affected in 26% (5). Additionally, up to 40% of index cardiac events are fatal in women (6). Thus, identifying effective preventive and diagnostic strategies is of particular importance.

CV risk in women is, however, underestimated because of the misperception that women are protected against ischemic heart disease. Lack of awareness of the importance of CV disease in women is widespread among general population and in the scientific community (7). In fact, younger women have especially low awareness rates despite high rates of CV disease burden and mortality (8). Underlying reasons for lack of awareness of women's CV risk include the following: (1) Women have been excluded from many CV clinical trials on the erroneous assumption that risk factor (RF) target values and imaging-based atherosclerosis phenotypes are similar in men and women (9); (2) Risk-stratification is especially challenging in women (8). Most women are classified at low 10-year CV risk, even in the presence of CV RF. Previous data demonstrates that all women aged 40–54 years had a low 10-year European SCORE risk (5, 10) while presenting high atherosclerosis prevalence and progression (3-year rate up to 30%) (11). Thus, although women's individual risk might be significant, they are less likely to receive guideline-indicated imaging screening or lifestyle recommendations (12); (3) Heart-disease risk in women is influenced by female-specific factors, such as age at menopause and hypertensive disorders of pregnancy, which are associated with accelerated atherosclerosis and impaired CV disease-free survival (13). However, these factors are not included in traditional risk assessments, and it is unknown if they improve risk classification independently of traditional RFs; (4) Atherosclerosis in women is under-recognized because female CV disease may have different clinical manifestations compared to men, resulting in suboptimal management. These observations highlight the need for women-specific approaches to increase awareness about CV risk and prevention.

In this article we describe the rationale and design of WAKE UP, a study aimed to improve CV disease awareness in women and healthy lifestyle promotion by using vascular ultrasound (VUS). Increasing awareness through noninvasive imaging may represent a new paradigm to bridge the gap because it reveals the presence of atherosclerotic plaques in a directly relatable way. The approach to CV disease management to date has been “inside in”, aimed at reducing RFs to minimize effects. WAKE UP is an “inside out” approach, whereby direct imaging of arterial health will be translated into specific lifestyle recommendations.

## Hypothesis and objectives

Misperception of the burden of CV disease in women is a barrier to achieving reductions in RF and atherosclerosis in women. Risk awareness will induce heart-healthy living and facilitate early recognition of symptoms. Our hypothesis is that the use of VUS will increase women's awareness, producing a greater impact on lifestyle than standard risk assessment. We predict that compliance with lifestyle recommendations would be higher among women who have undergone an imaging study because they will have seen visual evidence of their own vessel wall damage or plaque presence. This prediction is supported by the success of large evidence-based strategies that have already been conducted to promote CV health in adults and children. For example, in the TANSNIP trial, authors found that in middle-aged asymptomatic adults, a lifestyle intervention was associated with a significant improvement in CV health and behavioral metrics (14). Similarly, primordial prevention strategies in children based on school educational interventions have been shown to improve health outcomes (15). However, specific studies exploring this approach in women are lacking (16). Additionally, the identification of accurate predictors of atherosclerosis in apparently low-risk women who already have disease will help estimate individual CV risk and implement risk-reduction programs. We also aim to analyze other woman specific factors such as hormonal changes, complications during pregnancy or psychosocial factors that can influence the development of atherosclerosis. Moreover, analysis of complex data relations in a high volume of data is not an easy task, and novel data processing techniques are required.

## Methods

### Study design and population

WAKE UP is a longitudinal, prospective, case-control study that will include 720 women aged 40–70 years-old (from pre- to post-menopause) with at least one CV RF, defined as cigarette smoking, hypertension, dyslipidaemia, diabetes, obesity, poor diet, physical inactivity, family history of premature CV disease, metabolic syndrome, systemic autoimmune collagen-vascular disease, history of premature menopause, gestational diabetes or hypertensive disorders of pregnancy according to prevention guidelines for women (17). Exclusion criteria include any prior history of CV disease (myocardial infarction, angina pectoris, arrythmias, stroke, peripheral vascular disease, angioplasty, valvular heart disease, congenital heart disease or heart surgery), pregnancy and refusal to sign informed consent.

**Figure 1:**
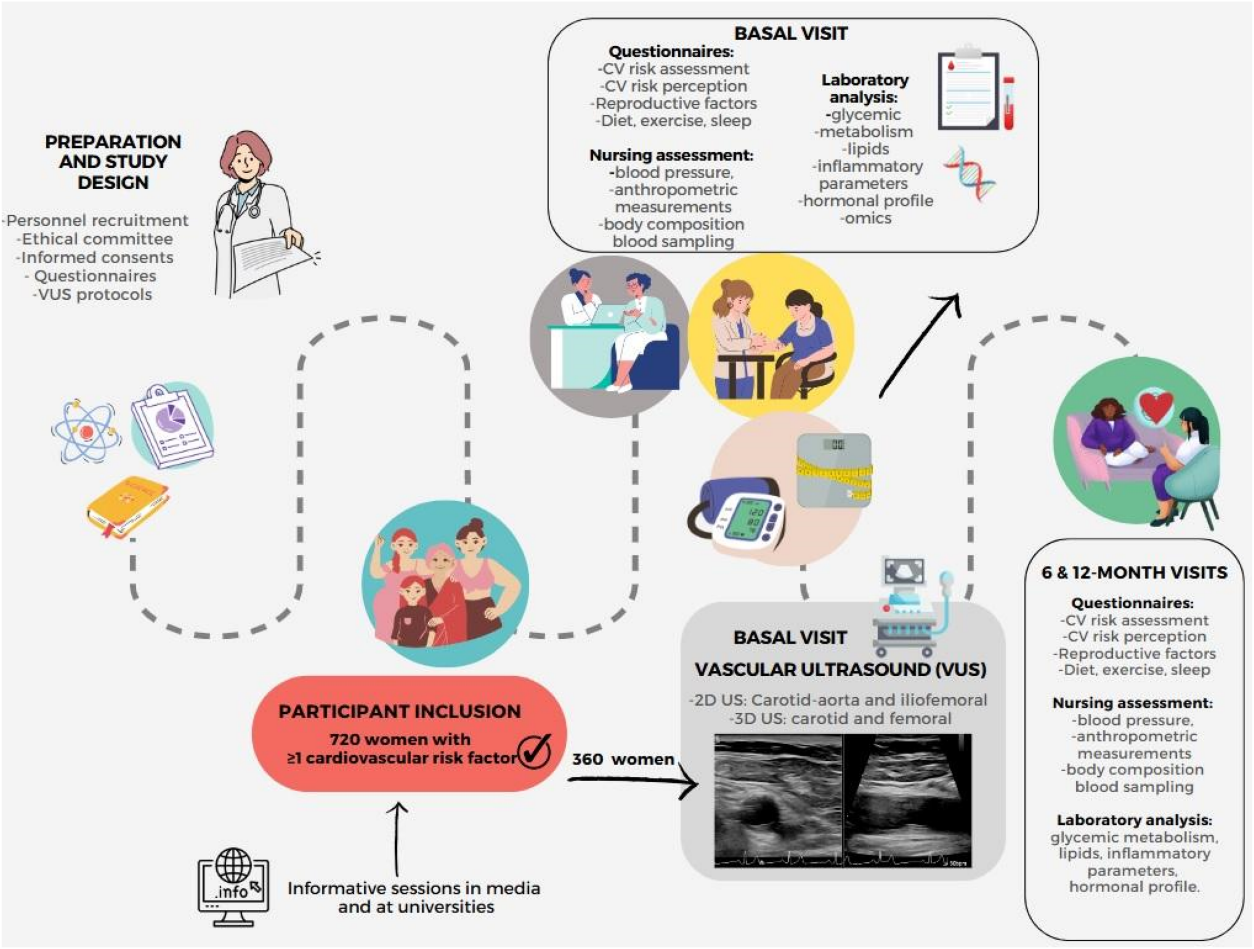
Central figure: WAKE UP study flow chart. CV, cardiovascular; US, ultrasound.

The study will include baseline, 6-month and 12-month follow-up visits (Figure 1). All visits will include traditional and non-traditional risk assessment, blood sampling and omics analysis (Table 1). Vascular imaging studies will be only performed at baseline, after randomization 1:1 for imaging risk assessment, 360 with VUS vs. 360 age- and RF-matched controls without imaging. Participants in the imaging group will be informed about their personalized CV risk based on imaging results and will be given lifestyle recommendations accordingly. Women with atherosclerotic plaque will be shown their arterial images and will be explained the meaning and CV risk associated with these findings. Those without plaques will be shown example images of atherosclerotic plaque and similar explanations will be given.

**Table 1:** WAKE UP study protocol and tests included in each visit.

**Procedure**	Baseline visit	6 and 12-month follow-up visits
CV risk factors	✓	✓
CV risk scores	✓	✓
Questionnaires	✓	✓
Anthropometric measurements	✓	✓
Blood sampling	✓	✓
Omics	✓	✓
Vascular ultrasound	✓	

CV, cardiovascular.

To ensure rigorous matching in the 1:1 randomization process, participants will be stratified based on key CV risk factors prior to randomization. These factors include age, body mass index (BMI), blood pressure, lipid profile, smoking status, and family history of CV disease. Stratified randomization will be implemented to achieve balanced allocation across the imaging and non-imaging groups while accounting for these variables. Randomization will be performed using a computer-generated sequence to eliminate selection bias, with allocation concealed until the intervention assignment. By controlling for these baseline characteristics, we aim to minimize confounding and ensure comparability between groups, thereby strengthening the validity of the study's findings.

The study will be conducted in 8 Hospitals, including HM Hospitales, Hospital Universitario Fundación de Alcorcón, Hospital de Toledo, Hospital Universitario Doce de Octubre in Madrid, HM Universitario Regla in León, Hospital Universitario Marqués de Valdecilla in Santander, H. Universitario de Cabueñes in Gijón y AtriaClinic in Madrid. Core Lab for vascular imaging studies will be Atria Clinic in Madrid, and the omics analysis for Madrid will be performed in CNIC (Centro Nacional de Investigaciones Cardiovasculares Carlos III). Recruitment will be promoted at the Hospitals, Universities, Spanish Society of Cardiology and media through appropriate material distributed by different dissemination channels (informative sessions, TV, journals, etc.). We are planning to organize meetings for public and dissemination of results in means of communications to expand awareness' of CV disease in women in Society. The study protocol has been approved by the Ethics Committees of Instituto de Salud Carlos III and HM Hospitals in Madrid. Written informed consent from all participants will be obtained and archived.

### Clinical data collection and risk scores

Each woman will undergo assessment of blood pressure, weight and body composition as well as a 1-h interview including all study questionnaires. A standardized 38-item questionnaire with a mix of Likert scale, open-end, and recognition questions will be used to assess CV risk perception. Similar to the American Heart Association National Survey (18), questions will be divided into 4 sections: general awareness of women's health issues; communications and behaviours related to CV disease prevention; specific understanding of CV disease and behaviours associated with prevention; and demographic characteristics. Smoking will be assessed by adults' tobacco use questionnaire from the National Health Interview Survey (NHIS) (19).

Diet will be assessed by a 14-item MEDAS (Mediterranean Diet Adherence Screener) questionnaire, used in the PREDIMED study that analyzes the consumption of the main components of the Mediterranean diet and their recommended amounts (20). Physical activity will be assessed by International Physical Activity Questionnaires (IPAQ) short version that assesses the types of intensity of physical activity and sitting time (21). Psychosocial and sleep aspects will be collected by the CES-D (Center for Epidemiological Studies-Depression), a 20-item questionnaire aimed at identifying individuals at risk for clinical depression with good accuracy across wide age ranges (22) and by a 12-item questionnaire that includes sleep habits, quality of sleep, and sleepiness (23). Women will be also asked about reproductive factors (age of menarche and menopause, contraceptive use, pregnancy, preeclampsia, gestational diabetes, preterm birth), family lifestyle (educational background, perception of family member medical conditions) and non-CV disease.

Several scores will be calculated to assess CV risk: Fuster BEWAT score (FBS) (24) compiles 5 variables (blood pressure, exercise, weight, alimentation, and tobacco) into a 15-point ordinal scale. For each variable, a numeric grading system (0, 1, 2, or 3) has been adopted in relation to international guidelines, being 3 the optimal value. Systemic Coronary Risk Estimation (SCORE) algorithm will be used to estimate risk of fatal CV disease by gender, age, systolic blood pressure, total cholesterol and smoking status (25). SCORE2 is an update of the original SCORE algorithm derived, calibrated, and validated to predict 10-year risk of first-onset CV disease by enhancing the identification of individuals at higher risk of developing CV disease across Europe (26). Atherosclerotic CV disease (ASCVD), based on age- and sex-specific pooled cohort equations estimates 10-year risk of nonfatal myocardial infarction, coronary heart disease death, or stroke for asymptomatic adults 40–75 years of age (27). Ten- and 30-year Framingham Risk Scores (28, 29) predict risk of hard coronary heart disease (coronary death, myocardial infarction, stroke).

### Laboratory analysis

Blood samples will be drawn for complete blood count, glycaemic metabolism (glucose and HbA1c), lipid panel (HDL-C, LDL-C, total cholesterol, triglycerides, lipoprotein A, apo B, apoA), thyroid function, ferritin and inflammation parameters (hs-CRP, leucocyte volume). Full hormonal profile will be analyzed making sure that premenopausal women are in the first 9 days of their menstrual cycle [estradiol, follitropin (FSH), lutropin (LH), sex hormone binding globulin (SHBG), free and total testosterone, dehydro-epiandrosterone sulfate (DHEAS), free androgen index (FAI), anti-Mullerian hormone].

Blood samples will also be processed and stored at −80°C for high-throughput “omics” analysis and biobanking by qualified personnel at CNIC. For epigenetics, DNA from the blood samples will be extracted using Infinium Methylation EPIC arrays, which include over 450.000 DNA bases and identify positions and regions hyper and hypomethylated for each time point to estimate the biological age using the getClock R package.

For lipidomics, plasma samples will be diluted with acidified methanol and lipids will be extracted with methyl-tert-butylether (MTBE) (30). Internal standard mixture containing Splash Lipidomix Mass Spec Standard and Ceramide C17 (d18:1/17:0) (Avanti Polar Lipids) will be added to the samples for quantification purposes. Organic phase will be dried-out, resuspended in appropriate solution and infused via nanoflow ESI source (Advion Triversa Nanomate) into a Quadrupole-Orbitrap Hybrid mass spectrometer (QExactive, Thermo Fisher Scientific). Data will be processed using LipidXplorer software (31).

### Vascular imaging protocol

**Figure 2:**
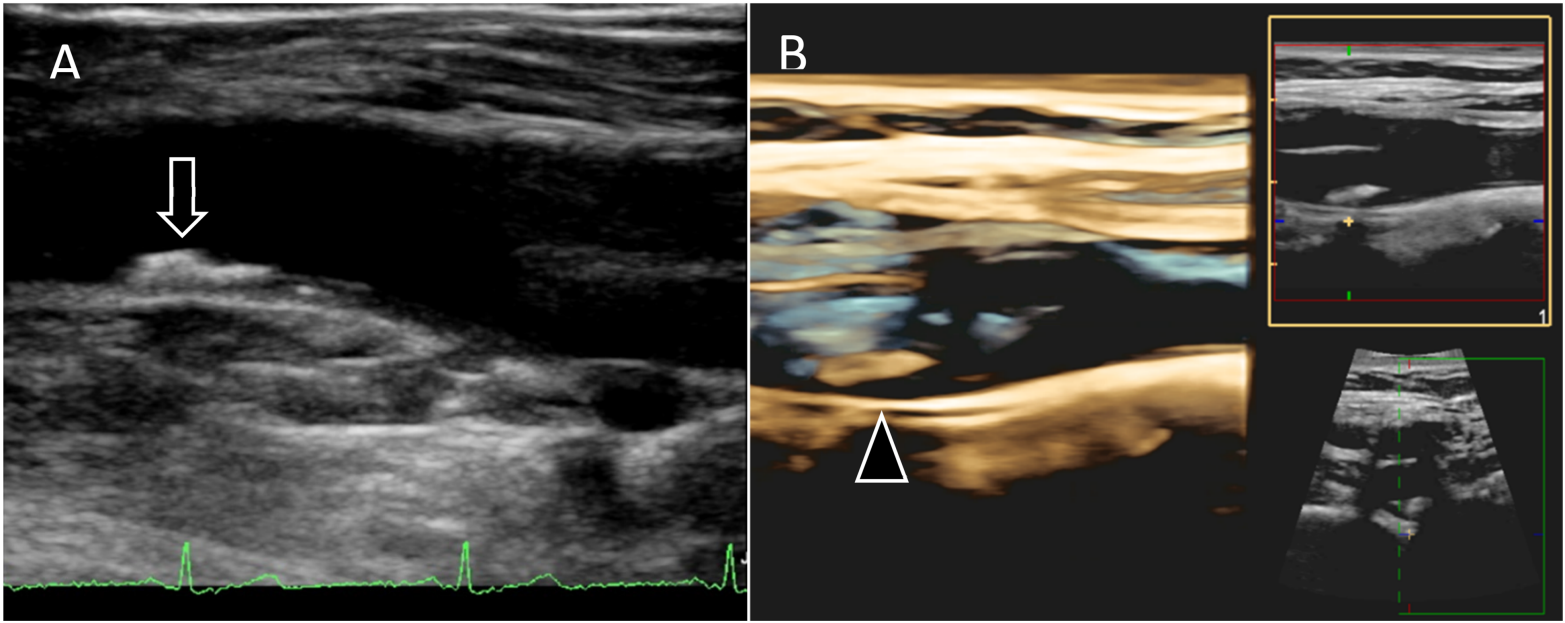
2D and 3D vascular ultrasound **(A)** 2D images of femoral artery showing atherosclerotic plaque (arrow) located in the common femoral artery prior to bifurcation. **(B)** 3D images of carotid artery bifurcation showing atherosclerotic plaque (arrow head) located in internal carotid artery.

**Figure 3:**
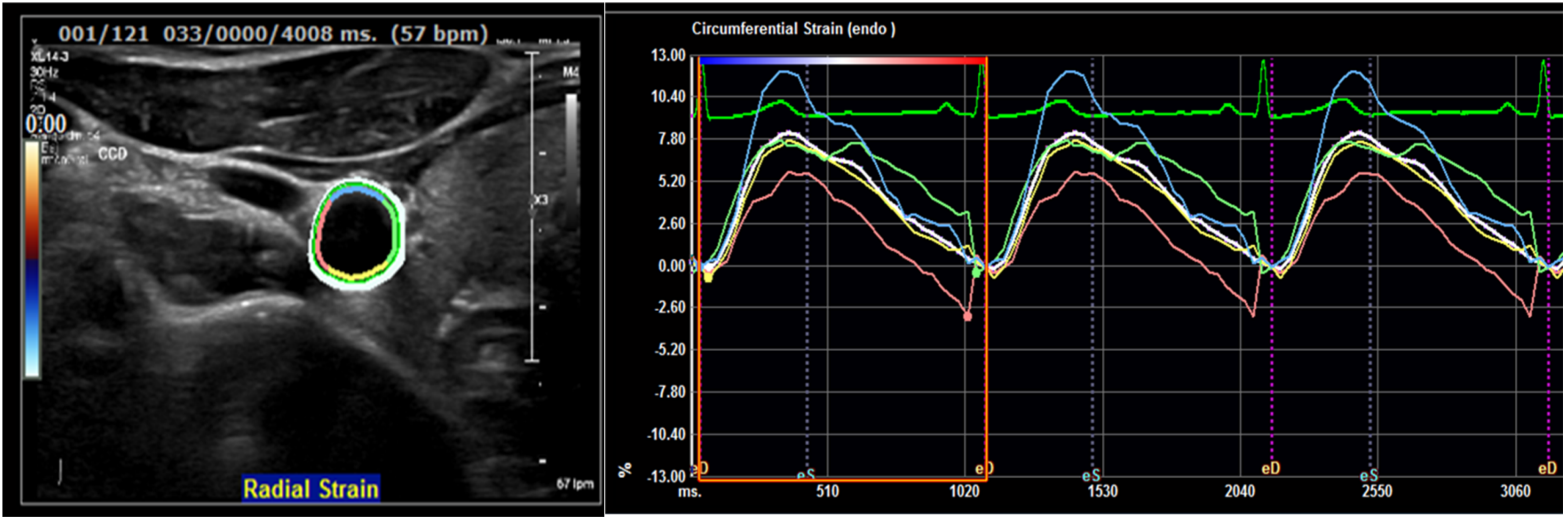
Carotid arterial strain curves with different colors representing the four arterial segments included in strain analysis the white line represents average radial strain.

Vascular ultrasound imaging will be carried out using Philips EPIQ Elite 7.0.5 and the protocol includes 2D VUS of the carotid, aorto-iliac and femoral territories (cross-sectional and longitudinal images) and 3D VUS of any plaque (volume set) with an approximate examination time of 40 min (Figure 2). Imaging analysis will include plaque presence, thickness, volume and degree of stenosis (5). Strain-rate measurements of the carotid cross-sectional image will also be assessed to increase our understanding of the elastic properties and function of the arterial walls providing potential information on arterial health status by analyzing distensibility/compliance coefficients, b-stiffness index, circumferential strain/strain rate, peak radial velocity (Figure 3) (32).

### Follow-up and end-points

At 6-month follow-up, participants will be contacted by telephone or email to undergo similar testing as baseline, except vascular imaging. The primary endpoint will be changes from baseline Fuster BEWAT score (FBS) which has shown a strong inverse association with subclinical atherosclerosis (24). FBS will enable the monitoring of global changes in healthy behaviors and non-laboratory-based CV health factors. The FBS measures CV risk exclusively on the basis of the easily measurable predictors blood pressure [B], exercise [E], weight [W], alimentation [A], and tobacco smoking [T], and is thus a highly relevant outcome given the characteristics of our study population. Secondary outcomes will include changes in overall knowledge, attitudes, and physical activity, diet, smoking, cholesterol, glucose, blood pressure, weight, and self-rated family health. We expected that women who underwent imaging will improve their CV lifestyle in a better way than women who do not undergo imaging because they will be witnesses of their own vascular damage, and thus, the FBS will score higher in the imaging group. Similar procedures will be carried out on the 12-month visit to evaluate long-term effects. CV events will be also registered in all follow up visitsfor participants with plaques, and also for those without plaques, as well as data regarding the approach taken by the participant inquiring whether she has any new medical treatment, has seen a healthcare professional with her results or if additional testing has been ordered based on blood tests or vascular imaging results.

### Statistical analysis

Preliminary descriptive statistical analysis (boxplots, histograms, univariate parametric and non-parametric tests) will be used once significant amounts of data have been collected. All parameters to be collected are summarized in Table 2. We will evaluate lifestyle changes by the FBS using multivariate logistic regression. The dependent variable will be the improvement or worsening of the score between baseline and follow-up, and the analysis will be adjusted for covariates such as age to identify groups of women potentially more impacted by the imaging-based strategy. Additionally, machine learning techniques will be derived to acquire knowledge from the data. In particular, supervised and semi-supervised classifiers will be trained with 80% of the data to derive risk prediction models. Remaining 20% of the data will serve as a validation cohort. Also, clustering analysis will be performed in order to observe phenotype groups in our patients that explain the clinical variance for sample size calculation, the following formula was applied:

$$n=\frac{{\left(Z_{\alpha /2}+Z_{\beta }\right)}^{2}\cdot (\;p_{1}\cdot (1-p_{1})+p_{2}\cdot (1-p_{2}))}{{\left(\;p_{1}-p_{2}\right)}^{2}}$$

**Table 2:** WAKE UP study variables.

Questionnaires	
- CV risk perception	health problems, personal health conditions, CV risk and general knowledge regarding CV disease in women
- CV risk factors	smoking, hypertension, dyslipidaemia, diabetes, obesity, poor diet, physical inactivity, family history CVD, metabolic syndrome, systemic autoimmune collagen-vascular disease, preeclampsia or gestational diabetes
- Non-CV disease	Thyroid disorders, chronic renal failure, pulmonary disease, hepatic disease, cancer, autoimmune disease
- CV risk scores	FBS, SCORE, SCORE 2, ASCV, FHS 10, FHS 30
- Smoking	Age of initiation, n° of cigarettes per day, exposure to secondary smoking
- Physical activity	Hours sitting on a workday, time spent walking on a workday, practice of moderate and/or intense exercise
- Diet	Portions of carbohydrates, proteins, fruits and vegetables, dairy products, sweets, fat, water and alcohol
-Reproductive factors	Age of menarche, contraceptive use and type, complications during pregnancy, age of menopause
-Psychosocial factors	Education level, employment status, marital status, stress scale, emotional state scale
-Family health (first-degree relatives)	Perception of family lifestyle, diet, exercise. Family history of CV risk factors, cancer, hepatic disease, thromboembolic disease
- Sleep	Sleep disorders (apnea, insomnia, restless legs syndrome, etc.) hours of sleep per night, difficulties in falling asleep or awakenings during the night
Anthropometric measurements	
Blood pressure, weight, height, BMI, waist circumference and body composition	
Biochemistry	
Glycemic metabolism	Glucose and HbA1c
Lipid panel	HDL-C, LDL-C, total cholesterol, triglycerides, lipoprotein A, apo B, apo A
Thyroid function	TSH, T4
Inflammatory parameters	hs-CRP, leucocytes volume, ferritin, albuminuria (urine sample)
Hormonal profile	Estradiol, follitropin [FSH], lutropin [LH], sex hormone binding globulin [SHBG], free and total testosterone, dehydro-epiandrosterone sulfate [DHEAS], free androgen index [FAI], anti-Mullerian hormone
Omics	
Lipidomic profile	Glycerophosphocholines, glycerophosphoethanolamines, glycerophosphoserines, glycerophosphoglycerols, glycerophosphoinositols, glycerophosphates and respective lysolipids; ceramides and sphingomyelins; diglycerides and triglycerides
Vascular ultrasound	
2D ultrasound: Carotid, aorto-iliac and femoral territories.	Plaque presence, no. of plaques, thickness of plaques, length of plaques and degree of stenosis
3D ultrasound: Carotid and femoral	Plaque volume and grey scale for composition
Vascular strain: Carotid cross-sectional image.	Distensibility/compliance coefficients, b-stiffness index, circumferential strain/strain rate, peak radial velocity

CV, cardiovascular; FBS, Fuster BEWAT score; SCORE, Systemic Coronary Risk Estimation; ASCV, Atherosclerotic CV disease; FHS, Framingham risk score.

*Z_α_*_/2_ and *Z_β_* are the critical values for the chosen significance level and power (splitting the significance level evenly between tails); *p*1 is the proportion of individuals who develop the disease without treatment, 45% according to the PESA study. *p*2 is the proportion of individuals who develop the disease with treatment, assuming 54% of our individuals will follow the provided recommendations to reduce the CVFR probability. With this setup, *n* = 481 patients will report a statistical power 1−*β* of 80% and significance level *α* of 95%, which are satisfactory in terms of clinical purposes. Given our previous experience with lifestyle intervention programs, we are well powered to detect relevant changes in the imaging group. Larger effects are anticipated in individuals with plaques, as there is more room for improvement. To account for potential variability introduced by including women across a wide age range, we will stratify participants into predefined age groups (e.g., young: 40–50 years: adult: 50–60 years, elderly: 60–70 years) during analysis. Additionally, we will apply multivariable regression models adjusted for age as a continuous variable to control for age-related confounding factors. This approach ensures that the impact of age on the progression of the atherosclerotic process is appropriately considered and mitigates the risk of contamination of information between age groups.

## Discussion

WAKE UP study is designed as part of a long-term global vision for improving women's health. Main goals are to promote women's CV health at different ages (pre- and post-menopause) and encourage appropriate lifestyle changes by increasing awareness through innovative strategies on CV disease prevention. Even though nearly 50% of women will die of CV disease, women's health priorities lie elsewhere (17). Lack of awareness results in suboptimal CV health prioritization and inaccurate risk assessment that ignores female-specific risk factors. Thus, there is the need for female-specific approaches and interventional programs to increase awareness of CV disease risk and prevention.

Noninvasive imaging allows the detection of early atherosclerosis, which improves risk reclassification and independently predicts CV events. The clinical significance of early atherosclerosis has been established in several large population-based studies, including MESA, HRP, Rotterdam and CAFES-CAVE studies (33–37). These studies showed that the presence of plaque, even in asymptomatic phases, was associated with CV events in long-term follow-up and improved risk reclassification, highlighting the importance of imaging early atherosclerosis (38). In the PESA study (39), which included >4,000 asymptomatic individuals, imaging was used to study multiterritorial subclinical atherosclerosis creating the basis for the inclusion in the latest European Guidelines of VUS imaging for atherosclerosis screening in low to moderate risk asymptomatic individuals (38). Vascular ultrasound imaging provides visual evidence of an individual's arterial health status and, more importantly, presence of plaque; this is especially attractive for raising awareness of the importance of CV disease in women and encouraging appropriate lifestyle changes. There are several trials that have explored the impact of visualization of coronary calcium score findings on the improvement of patient adherence to preventive care. Rozanski et al. compared the clinical impact of conventional risk factor modification to that associated with the addition of coronary artery calcium scanning finding that calcium scanning was associated with superior coronary artery disease risk factor control and improvement of patient adherence to preventive care (40). Another example of the effect of visualizing coronary calcium on improvement of statin treatment adherence was described by Kalia NK et al. (41). Event prediction with VUS is comparable to coronary calcium score, while avoiding harmful exposure to ionizing radiation, an especially important consideration for young women. Further advantages of VUS include the technique's portability, low cost, innocuous profile and potential improvement over population-based risk scales. We will use 2DVUS for plaque detection as well as 3DVUS, to assess atherosclerotic plaque burden with the added advantage of volumetric quantification. We also plan to develop a novel VUS application for vessel-wall tracking based on strain-rate techniques to assess early vascular mechanics changes specifically in women with RFs or subclinical disease. This technique will allow us to assess arterial stiffness, which is a surrogate of structural and functional changes within arterial walls and a potential marker of target-organ damage. Several validated ultrasound-based techniques and arterial stiffness parameters are available; however, all of them have technical limitations regarding acquisition, measurement and interpretation (32, 42) which have precluded their clinical implementation. Hence, there is a clear need for improved methods. According to previous studies, a 12-month period seems to be insufficient to observe notable changes in plaque dimensions and thus, VUS has not been included at follow up. WAKE UP will provide a one-of-a-kind dataset unavailable from any other study and will likely set the basis for future research directions, including a long-term follow-up for assessing subclinical atherosclerosis progression or regression as a result of lifestyle improvement and eventually, CV events in a female-specific cohort.

The WAKE UP study possesses a number of unique features: (1) it is the first and only large population-based study in women from pre- to post-menopause to use 2D/3D VUS to increase CV disease risk awareness in women and promote lifestyle changes. (2) It aims to integrate non-invasive imaging data with in-depth analysis of alternative determinants underlying atherosclerosis (reproductive and social factors, diet, physical activity). Knowledge of not only which factors contribute to disease but also which patterns are associated with the presence of atherosclerosis can provide a roadmap for shared decision making by patients and clinicians, allowing more aggressive interventions to be reserved for those at higher risk of developing complications. We also expect to be able to capture key differences between young, adult and elderly women, providing insights into the temporal sequence of atherosclerotic development, the interplay of age-related factors, such as hormonal changes or CV risk factors and opportunities for early interventions. (3) It can serve as a breakthrough strategy to initiate the establishment of multidisciplinary Women Heart Units which to date are lacking in Spain. Thus, the result of this project can have a high impact in the cardiovascular prevention field. The identification of efficient approaches to prevention and/or early detection of such a prevalent disease would have an enormous impact at multiple levels, from increasing life expectancy and improving quality of life to strengthening the workforce, reducing healthcare costs, and preventing neurocognitive decline. WAKE UP will provide a one-of-a-kind dataset unavailable from any other study, and will likely set the basis for future research directions.

Nevertheless, study limitations must be noted: the detection of CV RF in young womenpopulation for imaging randomization could be considered challenging. However national health statistics show that in women aged 40–54 years, the prevalence of hypertension, diabetes, high cholesterol, smoking, obesity and sedentary lifestyle is 10%, 3%, 12%, 25%, 25%, 40%, and 2%, respectively and numbers are even higher with increasing age. Similarly, we might detect a low prevalence of plaques in the enrolled women, especially of young ages. Based on the PESA study results, we expect to identify evidence of subclinical atherosclerosis with approximately 35% of all carotid plaques and 28% of all femoral plaques identified in the study occurring in women aged 50–54 years, with potentially more disease found in older women. In fact, the evaluation of wall mechanics, might reflect unhealthy arterial status before plaque deposition, an especially attractive approach in the female student cohort. Another challenge is related to the promotion of lifestyle changes at different ages. However, lifestyle intervention studies using the FBS as an outcome powers us to detect meaningful changes, especially in diet and physical activity (24). Moreover, larger effects are anticipated in individuals with plaques, given the direct visual evidence of vessel wall damage or plaque presence. This prediction is supported by the success of large evidence-based strategies that have already been conducted to promote CV health in children and adults (PESA-TANSNIP) (14). A deliberate decision was made to include women from a wide age range (from 40 to 70 years) within the same study rather than dividing them into separate cohorts. This approach provides several benefits: first, it enables the investigation of the atherosclerotic process across the lifespan, offering insights into temporal patterns that may not be apparent in age-specific studies. Second, integrating data from all age groups allows for a more comprehensive understanding of the interplay between age-related biological changes, hormonal effects and the progression of the disease. Third, by using statistical techniques such as stratification and multivariable adjustments, the potential for confounding will be mitigated, while preserving the ability to explore cross-age interactions. Although dividing participants into separate groups could reduce variability within each group, it would limit the ability to investigate overarching trends and relationships between age, lifestyle changes and disease progression. Future studies could complement our findings by focusing on more narrowly defined age cohorts, building upon the broad insights provided here.

## Conclusions

WAKE UP involves multiple innovative actions addressing a major public health need and has the potential to improve women's CV health and motivate complementary and synergistic actions elsewhere. Vascular ultrasound imaging arises as an attractive tool for increasing awareness of the importance of CV disease and encouraging appropriate lifestyle changes.

## Data Availability

The raw data supporting the conclusions of this article will be made available by the authors, without undue reservation.
